# Innate Sensing of Chitin and Chitosan

**DOI:** 10.1371/journal.ppat.1003080

**Published:** 2013-01-10

**Authors:** Chelsea L. Bueter, Charles A. Specht, Stuart M. Levitz

**Affiliations:** Department of Medicine, University of Massachusetts Medical School, Worcester, Massachusetts, United States of America; Duke University Medical Center, United States of America

Chitin is the second most common polysaccharide found in nature. It is present in crustacean shells, insect exoskeletons, parasitic nematode eggs and gut linings, and in the cell wall of fungi. The deacetylated derivative of chitin, chitosan, is less common but is particularly evident in certain species of fungi, such as *Cryptococcus*, and the cyst wall of *Entamoeba*. How mammals sense and respond to these polymers is not well understood, and conflicting reports on their immunological activity have led to some controversy. Despite this, promising translational applications that exploit the unique properties of chitin and chitosan are being developed.

## What Are Chitin and Chitosan?

**Figure 1:**
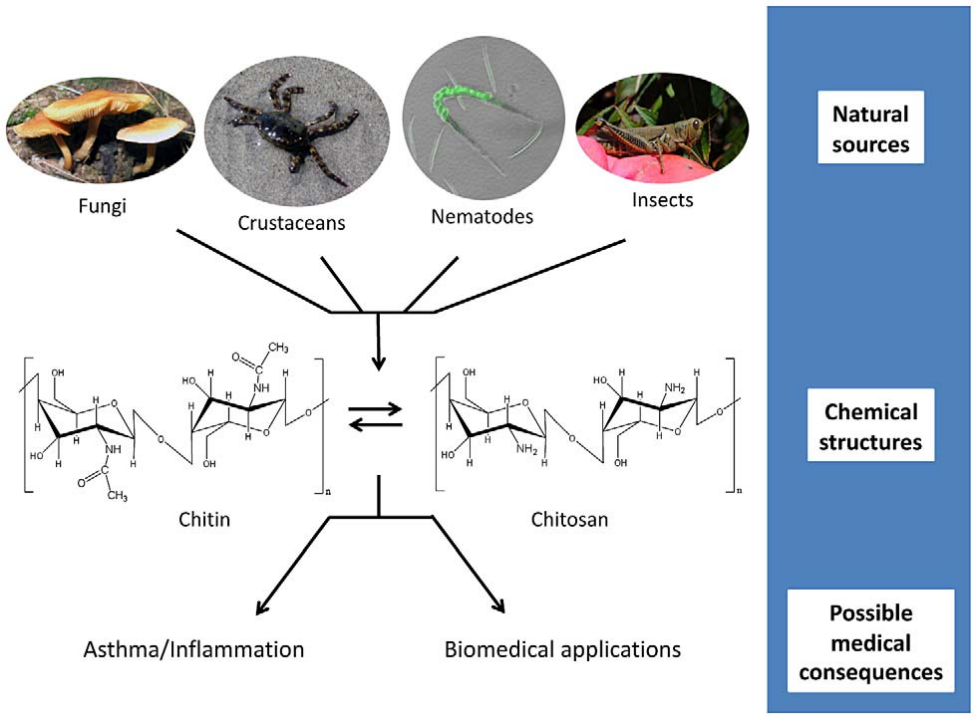
Chitin and chitosan: from source to consequence. Chitin and chitosan are naturally found in fungal cell walls, crustacean shells, nematodes eggs and gut linings, and insect exoskeletons. These polymers consist of long chains of N-acetylglucosamine (chitin) or glucosamine (chitosan). Conversion between the two polysaccharides can be performed chemically or happen within the organisms via chitin deacetylases. Mammalian exposure to the polymers has been linked to both upregulation and downregulation of inflammatory responses, including those involved in asthma. Despite this, chitin and chitosan are being utilized in a variety of biomedical applications, including tissue engineering and drug delivery.

Chitin, a linear, neutrally charged polymer of β-(1,4)-linked N-acetylglucosamine (GlcNAc), and its deacetylated derivative chitosan, a cationic polymer of glucosamine (GlcN), are two naturally occurring polysaccharides (Figure 1). Using cytoplasmic stores of UDP-GlcNAc, chitin synthases (EC 2.4.1.16) extrude chitin through the plasma membrane to an extracellular location [1]. Chitin deacetylases (EC 3.5.1.41), if present, remove the acetyl group following extrusion. During synthesis, chitin polymers anneal to one another, typically in opposite orientation (α-chitin), to form fibers of high tensile strength [2]. The fibers are cross-linked with glucans in fungi to form a meshwork reinforcing the cell wall and with protein in insect exoskeletons to give an ordered, laminate structure to the cuticle. Chitinases (EC 3.2.1.14) and chitosanases (EC 3.2.1.132) secreted by bacteria and fungi recycle gigatons of chitin/chitosan to GlcNAc/GlcN annually. In their more specialized roles, chitinases are important in developmental processes, such as remodeling the fungal cell wall and shedding old cuticle (molting) by crustaceans. For organisms that do not synthesize chitin, plant and mammalian chitinases aid in defense against chitin-bearing pathogens.

Commercial preparations of the polymers typically begin with isolation of chitin from crustacean shell waste, followed by chemical deacetylation to chitosan using high temperature and strong alkali. Particulate crystals of chitin are insoluble while chitosan is soluble in dilute acid. Commercial preparations of both polymers vary with regard to degrees of acetylation, polymer lengths, particle size, and contamination [3], [4]. Such heterogeneity likely accounts for some of the conflicting results in the literature.

## How Are Chitin and Chitosan Recognized by Mammals?

The lack of chitin or chitosan in mammalian cells makes these polymers potential targets for recognition by the innate immune system. Though chitin and possibly chitosan in their native environment can be stained with low molecular weight dyes, they are not readily accessed by protein-sized probes. Their exposure requires some degree of degeneration of their surrounding architecture, as occurs in damaged cells or fungal/crustacean detritus [3]. Upon exposure chitin can be recognized by mammalian chitinases, which bind and actively degrade chitin, and chitinase-like proteins, which also bind chitin but are catalytically inactive. It has become evident that this family of chitin-binding proteins (Glycosyl Hydrolase Family 18) plays an active role in inflammation and innate and adaptive immunity based on their upregulation during various disease states [5].

Both chitin and chitosan particles are readily phagocytosed [4], supporting a role for recognition via specific receptor(s) mediating phagocytosis. Receptors on myeloid cells that bind chitin or chitosan and induce a phagocytic response have yet to be definitively identified. However, several receptors have been shown to have an affinity for chitin or chitin oligosaccharides, including: FIBCD1, a homotetrameric 55-kDa type II transmembrane protein expressed in the gastrointestinal tract [6]; NKR-P1, an activating receptor on rat natural killer cells [7]; RegIIIγ, a secreted C-type lectin [8]; and galectin-3, a lectin with affinity for β-galactosides [9]. However, none of these has yet been shown to act as a receptor as opposed to a protein that binds chitin. Also, receptors that recognize soluble oligosaccharides as by-products of chitinase digestion may not recognize full-length, insoluble chitin.

## What Kind of Responses Does Chitin Elicit in Mammals?

Exposure to chitin, either through food or inhalation, is common. Chitin has been shown to induce a response similar to the response generated in helminth and allergic immunity, with an accumulation of eosinophils and basophils expressing IL-4, and alternatively activated macrophages [10]. Conversely, chitin downregulated the allergic response to ragweed in mice [11]. Also, asthma/allergic conditions feature alternatively activated macrophages, which have high expression levels of chitinases and chitinase-like proteins. Blocking acidic mammalian chitinase (AMCase) or knocking out BRP-39 (chitinase-like protein) results in decreased inflammation and eosinophilia [12].

Three innate immune receptors, Toll-like receptor (TLR) 2, Dectin-1, and the mannose receptor, have been implicated in mediating a variety of immune responses to chitin. However, how this occurs is not well understood. Direct binding to chitin has not been demonstrated, and the possibility that contaminants are responsible for some of these effects cannot be excluded. One study showed chitin acting via an apparent Dectin-1 dependent, but mincle (a C-type lectin), TLR2, and TLR4-independent mechanism could partially block cytokine production in response to *Candida albicans* [3]. Nevertheless, chitin was not shown to directly interact with Dectin-1. However, TLR2 was found to contribute to sensing of chitin by keratinocytes [13] and chitin-induced expression of IL-17A and IL-17AR [14]. Moreover, TNFα and IL-10 induced by chitin appeared to be mediated by TLR2, Dectin-1, and the mannose receptor. Interestingly, the size of the chitin particles determined the type of response observed: smaller fragments (<40 µm) induced cytokines that inhibited tissue inflammation, modest-sized fragments (40–70 µm) induced a strong pro-inflammatory response, and larger fragments were relatively inert [15]. Finally, though chitin preparations of varying sizes did not stimulate IL-1β production, chitosan was shown to activate the NLRP3 inflammasome, leading to robust IL-1β responses by a phagocytosis-dependent mechanism [4].

## How Do Plants Recognize and Respond to Chitin and Chitosan?

Fungi are major crop pathogens. It is not surprising then that plants exhibit a wide variety of defense responses to chitin and chitosan following fungal infestation, including increases in chitinase expression, proteinase inhibitors, reactive oxygen species (ROS), cytoplasmic acidification, and expression of early responsive genes and defense genes [16]. Presumably, most of these responses have developed to fight fungal infections, though chitin-binding lectins have also been shown to have insecticidal activity [17]. Likewise, fungi have developed methods to avoid recognition of chitin and thereby prevent the effective antifungal response, such as masking chitin with α-1,3-glucan, a compound plants are unable to digest [18]. Conversely, recognition of modified chitin oligosaccharides is important for symbiotic relationships between leguminous plants and rhizobial bacteria [19].

A number of receptors in plants that bind directly to chitin or mediate the response to chitin have been identified. Chitin elicitor-binding proteins (CEBiP) containing an extracellular lysin motif (LysM) that binds chitin directly are conserved across multiple plant species. CEBiP knockdown in suspension-cultured rice cells results in an absence of ROS generation in response to chitin [20]. CERK1, the *Arabidopsis* CEBiP homolog, is essential for chitin elicitor signaling; dimerization upon binding is critical for MAPK activation, ROS generation, and gene expression in response to chitin [21]. In contrast, chitosan appears to elicit activity from plant cells via charge-charge interactions with negatively charged phospholipids instead of via a receptor-specific interaction [22]. Whether analogous charge-based, receptor-free interactions between mammalian cells and this highly positively charged polymer occur is speculative.

## How Are These Polymers Being Used Translationally?

The unique structural and biological properties of chitin and chitosan are increasingly being exploited for use in biomedical applications, such as tissue scaffolds and wound dressings. This has been facilitated by advances in technology to produce purified polymers with desired physical properties. For example, particle size can be manipulated to control the resulting inflammatory response. The polycationic properties of chitosan are being developed for use in biosensors by immobilizing enzymes, in wound dressings to induce cell migration and proliferation at the wound site, and in tissue engineering as a scaffold [23].

The polycationic and biodegradable properties of chitosan make it attractive as a controlled delivery system for conjugated materials. Mucosal vaccines adjuvanted with chitosan have elicited robust antibody and T-cell responses [24]. Similarly, chitosan has been shown to have potential utility as a delivery system for drugs and genes [25]. Although there appears to be promising future applications for these polymers, currently chitin and chitosan are approved by the US Food and Drug Administration only for use as food additives. However, there are a number of ongoing clinical trials looking to expand their approved roles.

## Conclusions

Recent research has begun to clarify when and how mammals and plants recognize and respond to exposure to chitin and chitosan. Nevertheless, there are still many unanswered questions. Disparities in the literature regarding the immunological activity of chitin and chitosan are likely due in large part to the relative purity and heterogeneity of the glycan preparations used as stimuli. In particular, recent studies have demonstrated an inverse relationship between particle size and immunological activity. While much progress has been made in elucidating how plants recognize chitin and chitosan, the principal receptor(s) responsible for mammalian recognition remain to be determined. Finally, the biodegradable and physicochemical properties of chitin and chitosan make these glycans ideal for a wide range of translational applications.
